# A Growing Stem Inhibits Bud Outgrowth – The Overlooked Theory of Apical Dominance

**DOI:** 10.3389/fpls.2017.01874

**Published:** 2017-10-31

**Authors:** Tesfamichael H. Kebrom

**Affiliations:** College of Agriculture and Human Sciences, Prairie View A&M University, Prairie View, TX, United States

**Keywords:** apical dominance, auxin, shoot branching, internode elongation, sugar, source–sink relationship

## Abstract

Three theories of apical dominance, direct, diversion, and indirect, were proposed in the 1930s to explain how auxin synthesized in the shoot apex might inhibit axillary bud outgrowth, and thus shoot branching. The direct and diversion theories of apical dominance have been investigated in detail, and they are replaced with the current auxin transport canalization and second messenger theories, respectively. These two current theories still cannot entirely explain the phenomenon of apical dominance. Although there is ample evidence that the inhibition of bud outgrowth by auxin from the shoot apex is linked to stem elongation and highly branched auxin biosynthesis or signaling mutants are dwarf, the third theory proposed in the 1930s, the indirect theory, that explains apical dominance as auxin-induced stem growth indirectly inhibits bud outgrowth has been overlooked. The indirect theory did not propose how a growing stem might inhibit bud outgrowth. Recent discoveries indicate bud dormancy (syn. quiescence, paradormancy) in response to intrinsic and environmental factors in diverse species is linked to enhanced growth of the main shoot and reduced sugar level in the buds. Since a growing stem is a strong sink for sugars, and sugar is indispensable for shoot branching, the indirect theory of apical dominance might now be explained as auxin-induced stem growth inhibits bud outgrowth by diverting sugars away from buds. Detailed study of the indirect theory and the effect of source–sink status on dormancy and outgrowth of axillary buds will advance our knowledge of apical dominance and shoot branching in plants.

## Theories of Apical Dominance

**FIGURE 1:**
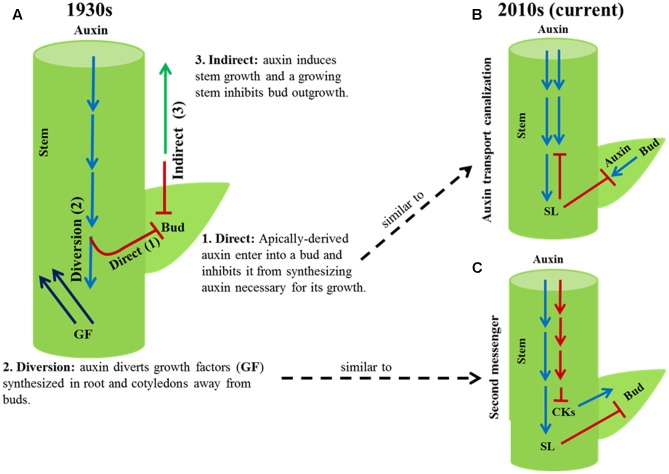
Old and current theories of apical dominance. **(A)** Three theories, direct (1), diversion (2) and indirect (3), were proposed in the 1930s to explain how auxin from the shoot apex might inhibit axillary bud outgrowth. The direct and diversion theories were investigated in detail and are now replaced with the current auxin transport canalization **(B)** and second messenger **(C)** theories, respectively. The indirect theory of the 1930s has been generally overlooked.

Plant shoot architecture often depends on the number of lateral branches or tillers developed and their position along the primary axis of the plant. A branch develops from a bud derived from a group of meristematic cells in the axil of a leaf. In some species, bud outgrowth is inhibited by signals from the apex of the main shoot, a phenomenon known as apical dominance. Soon after the discovery that the apical signal that inhibits axillary bud outgrowth is the plant hormone auxin (Thimann and Skoog, 1933), three theories – direct, diversion, and indirect (**Figure 1**) – were proposed to explain the phenomenon of apical dominance (reviewed in Snow, 1937).

The direct theory explains apical dominance as auxin synthesized in the shoot apex moves down the stem into buds and inhibits their growth. According to this theory, apically derived auxin inhibits buds from synthesizing their own auxin necessary for their growth. Through subsequent experiments the direct theory was disproved (Hall and Hillman, 1975; Morris, 1977; Prasad et al., 1993; Booker et al., 2003). It is now well established that auxin from the shoot apex inhibits bud outgrowth without entering into buds. However, the direct theory of the 1930s has some similarity to one of the two current models of apical dominance known as the auxin transport canalization (reviewed in Domagalska and Leyser, 2011). According to the auxin transport canalization model, auxin export from a bud into a stem is a necessary condition for bud outgrowth. The stem is saturated with auxin from the shoot apex; buds cannot export auxin into the stem, and thus become dormant (syn. quiescent, paradormant).

The second, diversion, theory of the 1930s explains the phenomenon of apical dominance as auxin from the shoot apex prevents growth promoting factors synthesized in roots and cotyledons from entering into buds and stimulate bud outgrowth. The diversion theory is similar to the current second messenger model, which proposes apically derived auxin inhibits bud outgrowth by regulating the level or activities of other plant hormones (Beveridge et al., 2009; Domagalska and Leyser, 2011). Consistent with this auxin inhibits the biosynthesis of cytokinin in the stem that stimulates bud outgrowth and promotes the biosynthesis of strigolactone in the roots that inhibits bud outgrowth (Beveridge et al., 2009; Muller and Leyser, 2011).

More than eight decades of research on the role of auxin in apical dominance has led to the formulation of the two current theories: auxin transport canalization and second messenger. However, the modes of action of auxin in apical dominance are not yet completely understood. For example, bud outgrowth in response to decapitation occurs prior to any change in the level of auxin in the decapitated stem (Morris et al., 2005; Ferguson and Beveridge, 2009). In addition, although inhibition of bud outgrowth by applying auxin to the stump of decapitated plants is dependent on strigolactones, auxin inhibits bud outgrowth in excised stems of strigolactone-deficient mutants (Young et al., 2014). Mutant analysis of some cytokinin-biosynthetic and response genes in Arabidopsis also questions the essential function of cytokinin in bud outgrowth resulting from decapitation (Muller et al., 2015). Application of auxin to decapitated stump of Arabidopsis and bean (*Phaseolus vulgaris*) does not fully restore apical dominance indicating factors other than auxin inhibit bud outgrowth in intact plants (Cline, 1996). Therefore, the auxin transport canalization and second messenger models cannot entirely explain the phenomenon of apical dominance (for detailed discussion, see Domagalska and Leyser, 2011).

## The Overlooked Theory Of Apical Dominance

The third, indirect, theory proposed in the 1930s explains apical dominance as auxin promotes the growth of the stem below the shoot apex, and the growth of the stem indirectly inhibits bud outgrowth (Snow, 1937). This third theory of apical dominance has been completely overlooked. Furthermore, although shoot branching is regulated by environmental and hormonal signals, the focus of research on shoot branching during the past 100 years was on apical dominance in eudicots such as pea because of the ease of application of hormonal treatments directly to the bud and measuring responses without damaging the plant (Beveridge et al., 2009). With the advent of modern physiological and genetic tools in recent decades, characterization of shoot branching mutants in diverse species has been useful in identifying genes and intrinsic and environmental factors regulating dormancy and outgrowth of axillary buds (Leyser, 2009; Kebrom et al., 2013; Rameau et al., 2015). The link between an increase in plant height and a reduction in shoot branching, and vice versa, in hormonal or light signaling mutants in diverse species (Beveridge, 2000; Stirnberg et al., 2002; Ishikawa et al., 2005; Kebrom et al., 2006; Simons et al., 2007; Finlayson et al., 2010) essential for exploring the indirect theory of apical dominance has been generally ignored. However, recent results demonstrating a link between stem internode elongation and inhibition of bud outgrowth (Kebrom et al., 2012) warrant reappraisal of the indirect theory of the 1930s because not only the phenomenon of apical dominance but also the regulation of shoot branching by intrinsic and environmental factors might be ultimately explained by the inhibitory effect of enhanced growth and elongation of the main shoot on axillary bud outgrowth.

It is well established that apically derived auxin promotes stem elongation and inhibits shoot branching, and highly branched mutants of auxin biosynthesis, transport or signaling pathways in diverse species are dwarfed. For example, plant height is strongly reduced and shoot branching increased in the *auxin resistant 1* (*axr1*) mutant of Arabidopsis (Lincoln et al., 1990). Reduced expression of the tomato auxin signaling gene *SlIAA15* concomitantly reduces plant height and increases shoot branching (Deng et al., 2012). Furthermore, auxin-overproducing *yucca* plants are elongated and display increased apical dominance. Transgenic reduction in the level of auxin suppressed the elongated primary shoot and increased apical dominance phenotype of *yucca* mutants (Zhao et al., 2001). Furthermore, shade signals promote shoot elongation and inhibit shoot branching (Franklin and Whitelam, 2005). The elongation growth response to shade is mediated through an increase in the biosynthesis, transport and signaling of auxin in leaves (Tao et al., 2008; de Wit et al., 2014; Procko et al., 2016).

**FIGURE 2:**
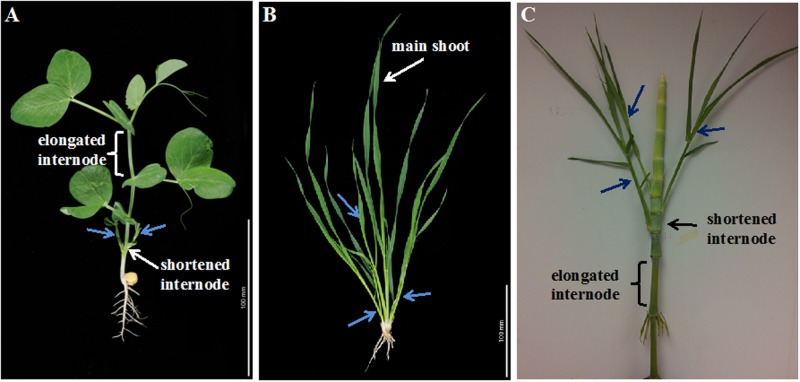
Stem growth and shoot branching in eudicots and monocots. **(A)** In eudicots such as pea, stem internodes elongate during the vegetative phase and shoot branching is inhibited. However, pea plants develop branches (indicated by blue arrows) from lower nodes adiacent to shortened internodes. **(B)** In monocots such as wheat, internodes do not elongate during the vegetative phase and tillers (basal branches, indicated by blue arrows) develop from the shortened internodes at the base of the shoot. **(C)** When grown at high density enriched with shade signals from neighbor plants, internodes of monocots such as sorghum elongate and bud outgrowth is suppressed. When the density is reduced internode elongation is suppressed and branches (indicated by blue arrows) develop.

It is also important to highlight that while apical dominance is often observed in eudicots such as pea it is not always apparent in grass species such as wheat (**Figure 2**). Plants grow through production of successive phytomers. Each phytomer has a leaf, a node, an internode and an axillary bud. In eudicots such as pea that display strong apical dominance, stem internodes elongate during the vegetative phase but buds do not grow (**Figure 2A**). When the growing shoot apex is decapitated, bud outgrowth begins immediately. A method known as shoot inversion in *Ipomoea nil* (morning glory) promotes bud outgrowth by suppressing the elongation of the stem section below the shoot tip (Hosokawa et al., 1990). In the grasses, stem internode elongation is suppressed during the vegetative phase and the shoot apex, enclosed by the sheath and young leaves, remains close to the base of the plant near the soil surface. During this phase tillers (basal branches) are formed and the tillering phase normally overlaps with the vegetative phase (**Figure 2B**). Once the shoot apex transitions to flowering phase, internodes begin to elongate and the tillering phase ends (McMaster, 2005). A more direct link between the inhibition of bud outgrowth by a growing stem has been identified in the tiller inhibition *tin* mutant of wheat (Kebrom et al., 2012). In *tin*, early cessation of tillering is associated with precocious internode elongation. In summary, there is ample evidence that supports the indirect theory that a growing stem inhibits bud outgrowth.

## A Growing Stem Diverts Sugar Away From Axillary Buds

The indirect theory of apical dominance proposed by Snow and others in the 1930s did not detail how auxin induced stem growth might inhibit bud outgrowth. Recent discoveries on the role of sugars in shoot branching in wheat, pea, sorghum, Arabidopsis, chrysanthemum, *Rosa* species, grapevine, and poplar provide new insights into the indirect theory of apical dominance (Kebrom et al., 2012; Rabot et al., 2012; Mason et al., 2014; Barbier et al., 2015; Kebrom and Mullet, 2015, 2016; Dierck et al., 2016; Tarancon et al., 2017). The inhibition of bud outgrowth in the *tin* mutant wheat is associated with precocious stem internode elongation and reduced sugar level in the buds (Kebrom et al., 2012). In pea, the sugar level in a dormant bud increases when the bud is stimulated to grow by decapitation that removes a growing shoot tip, which is a strong sink for sugars (Mason et al., 2014). In addition, dormant buds in intact pea plants grow when directly fed with sucrose providing conclusive evidence for the significance of sugars for bud outgrowth (Mason et al., 2014). Bud dormancy in the phytochrome B mutant sorghum (*phyB-1*) is associated with an increase in plant height and up-regulation of genes marker for sucrose deprivation in the buds (Kebrom and Mullet, 2016). Therefore, inhibition of bud outgrowth in the *tin* mutant wheat, pea and *phyB-1* sorghum is associated with enhanced growth of the main shoot and reduced sugar level in the dormant buds. Defoliation experiments in sorghum demonstrate that a small reduction in photosynthetic leaf area inhibits bud outgrowth while a more sever defoliation inhibits the growth of other sink organs including newly formed leaves in the main shoot (Kebrom and Mullet, 2015). This indicates, first, sugars could be limiting for plant growth in particular during the tillering/branching stages of plant development, and second, sink organs in the main shoot such as the stem and newly formed leaves are in a more favored position for sucrose than axillary buds. In the presence of strong sink organs such as a growing stem and limited sugar production in the main shoot, buds may become dormant. Therefore, the indirect theory of apical dominance can now be further elucidated as auxin-induced stem growth indirectly inhibits buds by depriving sugars necessary for their growth.

## Is Shoot Branching Determined by Source–Sink Status?

Apical dominance refers to the inhibition of bud outgrowth by the shoot apex. The dormancy versus outgrowth fates of axillary buds, and thus shoot branching is also controlled by other intrinsic and environmental factors besides auxin that act within or outside the bud (Leyser, 2009; Janssen et al., 2014; Rameau et al., 2015). A significant increase or decrease in plant height is commonly noticed in shoot branching mutants when the site of action of a gene is outside the bud. For example, strigolactones are synthesized primarily in the root, and almost all highly branched strigolactone biosynthesis mutants in diverse species are dwarf (Beveridge, 2000; Stirnberg et al., 2002; Ishikawa et al., 2005; Simons et al., 2007). The reduction in plant height in strigolactone mutants is not due to enhanced lateral branching (de Saint Germain et al., 2013). Since strigolactones promote internode elongation (de Saint Germain et al., 2013), it is possible that reduction in plant height in strigolactone deficient mutants stimulates shoot branching. In contrast, plant height and branching can be uncoupled when the site of action of the gene is in the bud. For example, the loss of function *teosinte branched1* (*tb1*) mutant of maize branch profusely while the height of the main shoot is not significantly different from the wild type (Guan et al., 2012). Mutation in the *tb1* ortholog *brc1* gene in Arabidopsis is non-pleiotropic and specifically increases shoot branching (Aguilar-Martinez et al., 2007). Interestingly, the expression of *tb1*/*BRC1* gene was found to be not sufficient for inducing bud dormancy (Kebrom and Brutnell, 2015; Seale et al., 2017). Furthermore, although cytokinins promote bud outgrowth when applied directly to the bud, buds in cytokinin deficient Arabidopsis plants grow in response to decapitation (Muller et al., 2015). Therefore, it appears that factors that control shoot branching by acting outside the bud override those that act within the bud and induce or inhibit bud outgrowth. As yet there is no known signal from the main shoot that is transmitted to the bud and controls its activity. However, sugar supply from the main shoot to the bud would be indispensable for bud outgrowth; the sucrose might also serve as a signaling molecule promoting bud outgrowth (Rabot et al., 2012; Mason et al., 2014; Barbier et al., 2015). Since an increase in plant height in response to environmental and intrinsic factors in diverse species is associated with a reduction in shoot branching, and dwarfism is associated with enhanced shoot branching, it is likely that shoot branching is determined mainly by source–sink status of the main shoot.

The plant source–sink relationship is a very complex process that depends on many factors including photosynthetic leaf area and efficiency, size and position of competing sinks, plant hormone dynamics and growth stage of the plant, and availability of nutrients such as nitrogen, light, and water (Lemoine et al., 2013; Albacete et al., 2014; Yu et al., 2015). For example, a small reduction in photosynthetic leaf area due to disease or herbivory could result in the inhibition of bud outgrowth in particular during the early stage of plant growth and development (Kebrom and Mullet, 2015). It is also possible that plants with relatively small photosynthetic leaf area at early stages of development such as Arabidopsis may not be able to develop branches during the vegetative stage. In sorghum, stem internodes are formed during the vegetative phase and elongate in response to high planting density or shade signals (Kebrom et al., 2017). As shown in **Figure 2C**, the length of internodes in a sorghum plant increased and reduced by alternating high and low plant density, respectively, and branches developed from buds adjacent to shortened internodes. In pea that displays strong apical dominance branches can still develop from buds in the lower nodes (Boyer et al., 2012), and unlike the elongated upper internodes, the lower internodes are shorter (**Figure 2A**). In maize, the length of internodes is negatively correlated to the number and size of ears that develop from axillary buds (Xu et al., 2004). Therefore, the size of internodes adjacent to the buds determines the sink strength of the internodes for sucrose utilization and storage, and indirectly regulates availability of sugars to the buds. However, a plant may grow taller and develop more branches when it synthesizes photoassimlates in excess. A concomitant reduction in plant height and shoot branching could also occur under poor growing condition. For example, Arabidopsis plants grown in low nitrogen are shorter and developed fewer branches than those grown at higher nitrogen (de Jong et al., 2014). In addition, mutations that reduce the overall growth of a plant might reduce both plant height and shoot branching. In fact, some of the plants reported as shoot branching mutants could be defective in the growth and development of the main shoot. For example, the reduced tillering (*tin*) wheat mutant is defective in the timing of development of internodes (Kebrom et al., 2012). Therefore, it appears that the tremendous variation in the number of branches and their position observed within and between species of annual plants could be in part due to variations in source–sink status of the main or parent shoots indirectly affecting the dormancy versus outgrowth fates of axillary buds.

## Summary and Future Perspectives

**FIGURE 3:**
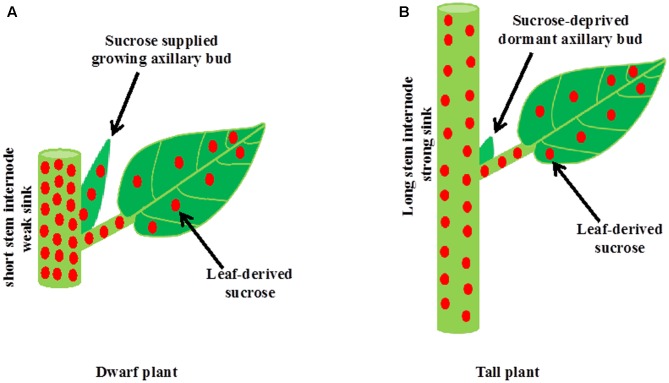
A model for the inhibition of bud outgrowth by a growing stem. **(A)** The growth of stem intemodes in auxin or strigolactone deficient mutant plants or plants grown at high light intensity or low planting density is suppressed. A short intemode is not strong sink for sucrose. Therefore, excess sucrose exported from photosynthetic leaves to the stem overflow into axillary buds and induces bud outgrowth. **(B)** Intrinsic factors such as auxin and strigolactones and environmental factors such as shade promote stem intemode elongation in the main shoot. Elongated intemode, which is a strong sink, inhibits bud outgrowth indirectly by limiting sugar supply to axillary bud.

It is well established that during apical dominance auxin from the shoot apex inhibits bud outgrowth indirectly without entering into buds. The two current theories of apical dominance, auxin transport canalization, and second messenger, describe processes in the main shoot in response to auxin from the shoot apex, including an increase in the level of strigolactones and a decrease in the level of cytokinins, leading to enhanced stem growth and formation of vascular tissues. Therefore, apically derived-auxin stimulates the growth of stem internodes in the main shoot and internode growth, which is a strong sink, inhibits buds indirectly by depriving sugars necessary for their growth (**Figure 3**). Intrinsic and environmental factors besides auxin that promote the growth and development of new sink organs including stem internodes and reproductive organs could also inhibit shoot branching indirectly by limiting sugars available for bud outgrowth. On the other hand, dwarfism in the absence of either auxin or strigolactones might stimulate shoot branching by making excess sugars available for growing buds. Therefore, shoot branching might be an unintended consequence of source–sink relationships and result from an overflow of sugars to axillary buds that cannot be utilized by the main shoot. While bud outgrowth depends on sugar supply from the main shoot, subsequent growth of the developing branch depends on an ample supply of nutrients and water from the roots. Nutrients are also one of the major factors determining the source–sink status, and thus indirectly regulate shoot branching.

The plant source–sink relations is regulated by intrinsic and environmental factors making shoot branching a complex trait that cannot be predicted easily without considering the growth and developmental status of the whole plant and prevailing environmental conditions. Reappraisal of the source–sink status in shoot branching mutants and wild-types and systematic study of the effect of source–sink status of the main shoot on dormancy and outgrowth of axillary buds might advance our knowledge of the physiological basis of apical dominance and shoot branching in plants. Future studies should accurately determine the sink or source status of an organ being manipulated. For example, the cotyledons in pea contribute to seed germination. The nutrient reserve and biomass of the cotyledons are exhausted within the first 10 days after sowing, during which the plant transitions from heterotrophic to autotrophic growth (Hanley et al., 2004). Experiments involving cotyledon removal or defoliation of young newly formed or old non-photosynthetic leaves assuming that they are source of nutrients or photoassimilates might lead to incorrect conclusions.

Besides their role in shoot branching, sugars are also important in many other aspects of plant growth and development including phase transitions from juvenile to adult and from vegetative to flowering (Wahl et al., 2013; Yang et al., 2013). Therefore, when investigating plant growth and development, sugar demand and supply should be taken into consideration.

## Author Contributions

THK conceived the idea and wrote the paper.

## Conflict of Interest Statement

The author declares that the research was conducted in the absence of any commercial or financial relationships that could be construed as a potential conflict of interest.
